# Resilience as a Mediator of Emotional Intelligence and Perceived Stress: A Cross-Country Study

**DOI:** 10.3389/fpsyg.2018.02653

**Published:** 2018-12-21

**Authors:** Ainize Sarrionandia, Estibaliz Ramos-Díaz, Oihane Fernández-Lasarte

**Affiliations:** ^1^Department of Personality, Assessment and Psychological Treatments, Faculty of Psychology, University of the Basque Country, Donostia-San Sebastian, Spain; ^2^Developmental and Educational Psychology Department, Education and Sport Faculty, University of the Basque Country, Vitoria-Gasteiz, Spain; ^3^Didactics and School Organization, Education and Sport Faculty, University of the Basque Country, Vitoria-Gasteiz, Spain

**Keywords:** emotional intelligence, resilience, perceived stress, undergraduate students, cross-country study

## Abstract

Existing literature provides evidence of the connection between emotional intelligence and resilience, both concepts being adversely related to perceived stress. Nevertheless, there is little evidence from cross-cultural and/or cross-country studies of the simultaneous relationship between these psychological variables. The objective of this study was to address this lack of research, examining the associations between emotional intelligence, resilience and perceived stress in a cross-country context. A total sample of 696 undergraduate students from two universities in the United States and the Basque Country (an autonomous community in northern Spain) participated in the study. Structural equation modeling was used to examine the effects of emotional intelligence and resilience that may affect students’ perceived stress. The results revealed that emotional intelligence functions as a negative predictor of perceived stress through the mediating variable resilience for the American and Basque students. The findings suggest that university students with better emotional intelligence and resilience present lower perceived stress. Thus, improving emotional intelligence and resilience could prevent students from suffering perceived stress in higher education. Implications and directions for further research are discussed; in particular, it is highlighted that intervention programs that improve both EI and resilience could be helpful in reducing perceived stress.

## Introduction

Since the first academic paper in 1990, research in emotional intelligence (EI) has grown considerably. However, very few cross-cultural/country studies have been carried out in the field. In general, these studies have found differences between European–American cultures and Eastern-Asian cultures, but not between European and American countries. Nozaki (2018) for example, found differences between European–American and Eastern Asian groups regarding the consequences of emotion regulation strategies. In particular, trait EI has been found to be negatively related to suppression (emotion regulation) in European–American groups, but not in the Japanese population. In the same vein, Gökçen et al. (2014) confirmed that there were cultural differences in trait EI. They compared two samples (Hong Kong and the United Kingdom) and they found that the British participants obtained higher EI compared to their Chinese counterparts. These findings were justified by explaining that the European-American population is individualist, while the Eastern Asian population is collectivist. In collectivist cultures, emphasis is placed on in-group achievement and interdependence, while in individualist cultures emphasis is placed on personal success and independence (Gökçen et al., 2014).

Resilience and perceived stress have also been examined in cross-cultural studies. However, results are contradictory. In a study comprising three different samples (United States, China, and Taiwan) it was found that resilience levels were similar in the different cultures (Li and Yang, 2016). Likewise, in a study conducted with Spanish and French students it was demonstrated that the results in relation to resilience were similar in both countries (Alonso-Tapia and Villasana, 2014). Regarding stress, in a cross-cultural study comprising three samples (Japan, Lithuania, and United States) it was shown that perceived stress differed across those cultures (Kononovas and Dallas, 2009).

Although EI, resilience and perceived stress have been examined separately in cross-country/cultural studies, there is not a single study investigating the relationship between the three variables across countries or cultures. Given that the University of the Basque Country is located in Europe and the University of Nevada in the United States, the two samples included in the study come from western (European–American) cultures, that is, collectivist cultures.

### Emotional Intelligence

Since the first scientific definition of EI in 1990, several theories have come up and currently there is no a single definition of the concept. The first scientific definition was provided by Salovey and Mayer (1990) who defined EI as “the ability to monitor one’s own and others’ feelings, to discriminate among them and to use this information to guide one’s thinking and action” (pp. 189). Later, in 1997, they modified this definition and presented a model with four branches (Mayer and Salovey, 1997; Mayer et al., 2004): Emotion perception, facilitation, understanding and regulation.

In the past 3 decades, several models have been developed, and EI is understood from different perspectives. On the one hand, EI can be considered an ability. On the other hand EI can be understood as a personality trait. Finally, EI can be taken as a mixed construct that comprise both abilities and personality traits. Regarding ability EI theories, ability EI is a cognitive ability related to the emotions that can be modified and improved thanks to intervention programs and trainings. As for trait EI theories, EI has been defined as a constellation of emotional perceptions located at the lower levels of personality hierarchies (Petrides and Furnham, 2001). Finally, according to mixed EI theories, EI is related to emotional abilities as well as personality traits.

This study is based on Mayer and Salovey’s (1997) four branch model: perception, facilitation, understanding and regulation of emotions. Perception of emotions refers to the ability to identify our own and other’s emotions, as well as the ability to identify emotions in other stimuli. Facilitation of emotions is related to the ability to use emotions to assist in certain cognitive enterprises, such as problem solving, interpersonal communication or reasoning. Understanding of emotions involves the ability to analyze emotions. Regulation of emotions involves the ability to modify an emotional response.

Emotional intelligence (EI) is related to many important life factors. In fact, EI is a significant predictor of subjective well-being (Andrei et al., 2016), job performance (O’Boyle et al., 2011), interpersonal relationships with romantic partners (Malouff et al., 2014), social support (Goldenberg et al., 2006), IQ (Webb et al., 2013) and health (Martins et al., 2010; Mikolajczak et al., 2015); while it is negatively related to loneliness (Anguiano-Carrasco et al., 2015) and depression (Webb et al., 2013), among others. Likewise, EI can be improved thanks to trainings and intervention programs (Mikolajczak and Peña-Sarrionandia, 2015).

### Resilience

After a stressful life event, some individuals have the capacity to recover more quickly than others and draw strength from the situation. Resilience has been described as a dynamic process where an individual adapts positively to an adversity (Luthar et al., 2000). That is, resilience is the capacity of a dynamic system to adapt successfully in the context of significant threats to system function, viability, or development (Masten, 2013).

Although there are different approaches to understanding resilience, in the present study resilience is considered as a trait. According to this approach resilience is a positive personality trait that promotes adaptation (Wagnild and Young, 1993; Connor and Davidson, 2003). In fact, resilience is considered as a series of individual attributes that can facilitate the ability to cope when confronted with stressful life events (Hoge et al., 2007). Based on a multidimensional nature of resilience, Connor and Davidson (2003) explained that there can be different reactions to a stressor. On the one hand, the stressor may represent a chance to grow and increase the person’s resilience, and thereby promote a come back to a higher level of balance. Conversely, the individual may have adjustment problems and deploy destructive means to cope with the stressor. This implies that resilient individuals could maintain their psychological health by buffering negative effects from difficult times.

Resilience has been associated with well-being (Harms et al., 2018), satisfaction with life, affect, self-concept and engagement (Sagone and De Caroli, 2014; Bajaj and Pande, 2016; Rodríguez-Fernández et al., 2016). Likewise, resilience has been found to be related to personal competence, high standards and tenacity; trust in one’s instincts, tolerance of negative affect, and strengthening effects of stress; positive acceptance of change, and secure relationships; control; and spiritual influences (Connor and Davidson, 2003).

### Perceived Stress

Subjective perception of stress continues to be a relevant concept of considerable interest in health studies. It is associated with a person’s overall health status and different diseases, including adjustment disorders (Vallejo et al., 2018). Understood as a maladaptive indicator, Lazarus and Folkman (1984, p. 19) defined stress as a “relationship between the person and the environment that is appraised by the person as taxing or exceeding his or her resources and endangering his or her well-being. Stressful events, and thus perceived stress, appear throughout the life cycle, and they also affect college students. Indeed, there is increasing empirical evidence for the presence of psychological problems in young adults, especially during their years at university (Milojevich and Lukowski, 2016). Undergraduate students are moving into and through a major developmental period of transition, and stress is becoming more prevalent among this population (Beiter et al., 2015). Consequently, they face different stressful situations due to the challenging developmental tasks of the young adulthood stage which can limit their psychological comfort. According to the previous research, university life can be considered as a potentially stressful situation and college students display high levels of psychological distress, such as depression, anxiety, and specially stress (Saleh et al., 2017b). Some studies have found that the tendency to experience unpleasant emotions and suffer from low self-esteem, little optimism and a low sense of self-efficacy could be stress predictors in college students (Saleh et al., 2017a). Notwithstanding the above, many studies also highlight the existing link between certain positive traits like individual differences and stress regulation processes (Nelis et al., 2009; Thomas et al., 2018).

### Relationship Between Emotional Intelligence, Resilience and Perceived Stress

In the following paragraphs, the results of studies evaluating the relationships between EI, resilience and perceived stress are presented. The first paragraph refers to the studies investigating the relationship between EI and resilience, the second paragraph focuses on EI and stress and the last paragraph presents the results of the studies that evaluating the relationship between resilience and perceived stress.

In relation to the association between EI and resilience, the vast majority of research in the area shows that people with better EI have better resilience. In particular, Schneider et al. (2013) demonstrated that EI facilitates stress resilience. In fact, the four EI abilities appeared to facilitate resilient stress responses including challenge appraisals, more positive and less negative affect, and challenge physiology. Likewise, Magnano et al. (2016) showed that EI plays a significant role on resilience. In the same vein, Armstrong et al. (2011) revealed that EI was related to psychological resilience. According to these authors, having higher EI is adaptive in stressful circumstances. Salovey et al. (1999, p. 161), for their part, confirm that people with better EI fare better with the emotional requests of stressful situations as they are able to “accurately perceive and appraise their emotions, know how and when to express their feelings, and can effectively regulate their mood states.” Finally, Cejudo et al. (2016) confirm that people with a high level of EI show a greater degree of resilience, being the correlation between emotion repair and resilience the most significant (among the different EI dimensions).

In terms of the relationship between EI and stress, the literature confirms that emotionally intelligent people show less perceived stress. According to Zysberg et al. (2017), stress levels mediate the association between EI and burnout. Likewise, Jung et al. (2016) found an inverse correlation between EI and self-reported stress. Similarly, Urquijo et al. (2016) suggested that EI enhances well-being, diminishing the experience of stress.

Finally, with respect to the link between resilience and perceived stress it is necessary to highlight that in the previous research resilience is clearly conceptualized as the ability to cope after a stressor (Masten, 2001; Connor and Davidson, 2003). Even common life stressors may require coping. Consequently, this study is based on the idea that resilience should reflect with the successful management of stressors in general (Seery and Quinton, 2016). As resilience implies the ability to recover from undesirable circumstances, it can protect one’s positive psychological functioning against stressors. In addition, previous studies suggest that psychopathological symptoms are closely linked with resilience (Southwick et al., 2005; Yu et al., 2016), so high resilience scores are generally associated with fewer indicators of maladjustment. To the contrary, lower levels of resilience forecast injured psychological functioning. Besides which, in the family context resilience has been negatively associated with adolescent/young adults’ perceived stress (Chen et al., 2018); however, it would be beneficial to examine this relationship thoroughly. Thus, there are limited data about the protective role of resilience on perceived stress in undergraduate students.

In short, it should be noted that although there are indeed some studies assessing the relationship between two of the three variables evaluated in the study (EI-resilience, EI-perceived stress, resilience-perceived stress), there is a huge deficiency regarding the relationship between the three variables (EI-resilience-perceived stress). In fact, there is not a single study assessing this relationship; and this gap should be closed.

### Aims of the Present Study

**FIGURE 1:**
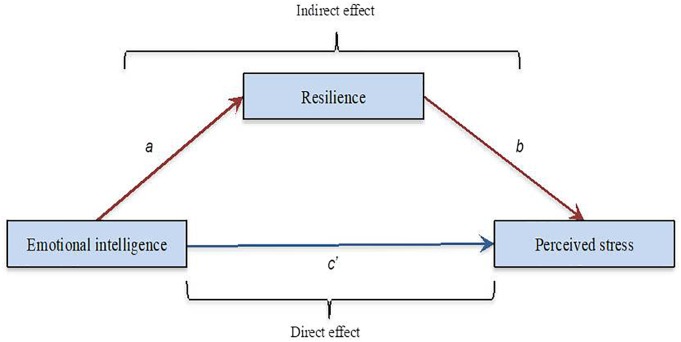
Hypothesized structural model.

Based on prior research in the field of EI, resilience and perceived stress, the present study had two main aims. Firstly, this study was aimed at exploring the relationships between EI, resilience and perceived stress in two different countries: America and Basque Country. The use of such samples would help to identify the cross-country replicability of the relationships between the variables of the study, but would also provide a first insight into specific associations within each group. Secondly, the study aimed to shed light on the way EI affects perceived stress by analyzing the potential mediating influence of resilience to bridge the gap in relation to previous research. Moreover, a better understanding of these associations would have a crucial practical impact concerning stress prevention. The model with EI as predictor, resilience as mediating variable and perceived stress is shown in Figure 1.

The following hypotheses were therefore formulated:

Hypothesis 1: EI positively predicts resilience.
Hypothesis 2: Resilience negatively predicts perceived stress.
Hypothesis 3: EI negatively predicts perceived stress.
Hypothesis 4: The effect of EI on perceived stress is mediated by resilience. In particular, EI should lead to higher resilience, which should in turn lead to lower perceived stress (high EI → high resilience → low perceived stress).
Hypothesis 5: The relationships between EI-resilience-perceived stress will be similar in both countries.

## Materials and Methods

### Design

We performed a cross-country comparative analysis of data from undergraduate student population in the United States and the Basque Country (Northern Spain). These two natural groups belonging to different cultures were compared at a given point in time. This is an explanatory design with latent variables in which structural equation models make it possible to test the effects of partial mediation as well to compare the adjustment of alternative full mediation models.

### Participants and Procedure

Participants were comprised of 698 undergraduate students (232 male, 466 female; *M*_age_ = 20.69 years, *SD* = 2.33; age range 18–45) enrolled in Psychology and Education faculties. Among these 698 students, 300 were recruited from the University of Nevada (United States) and 398 from the University of the Basque Country (Northern Spain), using the convenience sampling method. Response rate was 92% in the United States and 95% in the Basque Country.

The University of the Basque Country is the public university of the Basque Country. It has three campuses over three provinces receiving more than 40,000 students. The university offers 68 degrees, 111 official masters, and 65 Ph.D. programs. According to the Shanghai Ranking (ARWU), the University of the Basque Country is among the best 400 universities of the world (2018). The University of Nevada is a public research university located in Reno, Nevada. It has more than 18,000 students and offers more than 100 degrees, certificates and licensures in more than 145 academic majors. The university was ranked joint 197th among national universities by United States News and World Reports (2017).

A link to the online survey was given to American students, and data from the Basque sample was obtained using paper and pencil questionnaires. The demographic characteristics of the respondents can be found in Table 1 for both samples. Participation was strictly anonymous and voluntary. The students agreed to participate in the research via informed consent or mouse click in line with the questionnaire method. Participants did not receive financial compensation.

**Table 1:** Demographic characteristics of the study samples.

Country	Questionnaire method	Sample size	Males/females	Age
United States	Online	300	90/210	*M* = 21.56 (*SD* = 2.82)
Basque Country	Paper-pencil	398	140/256	*M* = 20.13 (*SD* = 1.72)
Total		698	230/466	*M* = 20.69 (*SD* = 2.33)

### Measures

#### Perceived Emotional Intelligence

This construct was evaluated using the Self-Rated Emotional Intelligence Scale (SREIS; Brackett et al., 2006). It is a self-rating measure with 19 items and a 5-point Likert-type scale (ranging from 0 = *very inaccurate* to 4 = *very accurate*) that allows the participants to describe, in their view, how accurate or inaccurate each statement is. The SREIS assesses in both oneself and others the perception of emotions (e.g., By looking at people’s facial expressions, I recognize the emotions they are experiencing), use of emotions (e.g., When making decisions, I listen to my feelings to see if the decision feels right), understanding emotions (e.g., I have a rich vocabulary to describe my emotions) and management of emotions (e.g., I can handle stressful situations without getting too nervous). The total SREIS score ranges from 1 (low emotional intelligence) to 95 (high EI). Brackett et al. (2006) conducted several studies in which the reported Cronbach’s alphas for the SREIS were as follows: 0.84 (study 1), 0.77 (study 2), and 0.66 (study 3). In this study, the full scale was reliable: 0.75 and 0.81 for the United States sample and Basque sample, respectively. As for the four dimensions that assess the SREIS, the reported Cronbach’s alphas are the following: Perception of emotions 0.70, Facilitation of emotions 0.67, Understanding of emotions 0.84, and Regulation of emotions 0.75.

#### Resilience

Participant resilience was measured with the 10-item Connor-Davidson Resilience Scale (10-item CD-RISC; Campbell-Sills and Stein, 2007). This self-report scale is an abbreviated version of the Connor-Davidson Scale (Connor and Davidson, 2003) that consists of 10 items (e.g., Can deal with whatever comes, Able to adapt to change) preceded by the following opening phrase: “In the last month, how often have you felt…”. Respondents rate themselves on a 5-point Likert Scale (ranging from 0 = *never* to 4 = *almost always*). The answers to the items are added up to create a resilience score (range 0–40), with higher scores indicating greater resilience. Psychometric evaluation of the 10-item CD-RISC conducted on undergraduate samples demonstrated that the scale had good reliability (Cronbach α = 0.85) (Campbell-Sills and Stein, 2007). In the present sample, the internal reliability indices of the 10-item CD-RISC were 0.83 for the United States sample and 0.73 for the Basque sample.

#### Perceived Stress

The Perceived Stress Scale 4 (PSS-4) was applied to evaluate the degree to which life situations are perceived as stressful (Cohen et al., 1983; Cohen and Williamson, 1988). This questionnaire asks participants about how unpredictable, uncontrollable, and overloading respondents find their lives, and how they think they felt during the last month. The 5-point Likert scale (ranging from 0 = *never* to 4 = *very often*) allows the respondent to agree or disagree with a series of statements. In particular, the four items are “*In the last month, how often have you felt that you were unable to control the important things in your life*?”, “*In the last month, how often have you felt confident about your ability to handle your personal problems?*”, “*In the last month, how often have you felt that things were going your way?*”, and “*In the last month, how often have you felt difficulties were piling up so high that you could not overcome them?*”. Total psychological stress score is ranged between 0–16, with higher scores suggesting higher psychological perceived stress. The four-item version of the PSS-4 presents good reliability and validity (Cohen et al., 1983). In the original study Cronbach’s alpha of this abbreviated scale was 0.72. In the present sample, the internal reliability indices of the PSS-4 were 0.70 for the United States sample and 0.70 for the Basque sample.

### Ethical Considerations

The study adheres to the ethical values set up for psychological research and assessment, and respected the basic principles arranged in the American Psychology Association’s ethics code and in current regulations (informed consent and the right to information, protection of personal data and confidentiality guarantees, non-discrimination, non-remuneration and the right to withdraw from the study at any time). The protocol was approved by the Ethical Committee for Investigations related to Human Beings (University of the Basque Country).

### Data Analyses

Descriptive statistics were performed using SPSS Statistics 24.0 and the confirmatory analysis was computed using SPSS Amos 24. There is not a high percentage of extreme values, which could distort further testing, and a decision was made not to disregard them. In fact, these extreme values are representative of the sample object of interest. The rate of missing data was low and therefore, no imputation procedures were implemented.

The bootstrap method was applied, as offered by the AMOS 24 program (with 2000 repetitions and establishing a confidence interval of 95%). This method calculates the empirical distribution for the statistics using random sampling with replacement. Therefore, the estimates are robust insofar as they are not affected by a lack of normality in the residual distribution.

Before being able to test the hypothesized model, the measurement portion of the model needs to be specified, so the two-step procedure (Anderson and Gerbing, 1988; Byrne, 2013) was followed in this study. According to this method, the task involved in developing and testing the structural model is twofold: The first step involves a confirmatory factor analysis (CFA) of the measurement model, which includes the relationships between the observed variables and the latent variables. The second step includes a CFA of the causal relationships between the constructs of the model as specified by the theory.

In order to verify the hypothesized model regarding the potential mediating role of resilience between EI and perceived stress, structured equation procedure was applied. The maximum likelihood estimation (ML) method was used. To examine the overall fit of the model to the data, several indexes proposed by Hu and Bentler (1999) and Kline (2015) were computed in the present study: chi-square statistic (χ^2^) and its level of associated probability, CFI (Comparative Fit Index), TLI (Tucker–Lewis Index), RMSEA (Root Mean Square Error of Approximation) with its confidence interval (CI), and SRMR (Standardized Root Mean Square Residual). The chi-squared test was also included to compare the estimated models. For the CFI, values over 0.90 suggest acceptable fit, while values over 0.95 suggest a good fit. Values on the SRMR and the RMSEA near 0.05 suggest an excellent fit, whereas values between 0.05 and 0.08 suggest an acceptable fit (Hu and Bentler, 1999; Kline, 2015).

## Results

### Descriptive Statistics and Relationships Between the Study Variables in the Two Samples

To examine the measurements (SREIS, CD-RISC 10, and PSS-4), mean, standard deviation and reliability analyses (Cronbach’s alpha) were computed. Furthermore, Pearson correlations between the three study variables were conducted in order to analyze the relationships between EI, resilience and perceived stress. The descriptive statistics, correlation coefficients, and Cronbach’s alphas for each of the two samples are presented in Table 2.

**Table 2:** Correlation coefficients, descriptive statistics and alphas for the United States and Basque Country samples.

	United States (*N* = 300)			Basque Country (*N* = 396)		
	
Measures	1	2	3	1	2	3
(1) SREIS	1	0.52^∗∗^	-0.29^∗∗^	1	0.39^∗∗^	-0.48^∗∗^
(2) CD-RISC		1	-0.48^∗∗^		1	-0.11^∗∗^
(3) PSS-4			1			1
*M* (0–4 scale)	3.36	3.13	2.11	2.92	2.65	2.16
*Sd*	0.44	0.51	0.60	0.44	0.43	0.42
Cronbach α	0.75	0.83	0.70	0.81	0.73	0.65

*^∗∗^p < 0.01. N_*American sample*_ = 300 (90 men, 210 women); M_*Basque sample*_ = 396 (140 men; 256 women); SREIS: Self-Rated Emotional Intelligence Scale; CD-RISC: Connor-Davidson Resilience Scale (10 item version); SPSS-24: Perceived Stress Scale (4 item version)*.

Table 2 displays the means of the SREIS in the Basque sample and the PSS-4 in both samples ranged around the scale midpoints (i.e., 47.5 for the SREIS; 8 for the PSS-4). Means of the SREIS in the American sample and the CD-RISC 10 in both samples were significantly above the scale midpoint (i.e., 20 for the CD-RISC 10 and 47.5 for the SREIS). Standard deviation showed the tendency to be higher in both samples. Internal consistencies were acceptable as showed by Cronbach’s alpha coefficients.

To analyze the relationships between EI, resilience, and perceived stress, correlations between the SREIS, CD-RISC10, and PSS-4 were estimated. While EI had positive and significant association with resilience, EI and resilience were negatively related with perceived stress. In the American sample resilience was more strongly related to EI and perceived stress than in the Basque sample. Besides which, a statistically significantly higher correlation coefficient was found in the Basque sample regarding the relationship between EI and perceived stress.

### Mediational Analyses

#### Measurement Model

The measurement model consisted of three interrelated latent variables (EI, resilience, and perceived stress), whose indicators, in the case of resilience and perceived stress, were those items of the questionnaires in the corresponding test. With regards to EI, parcels of items were used as manifest variable in structural equation modeling procedure. In accordance with the internal-consistency method, parcels were created by using the facets as the grouping criteria. Each parcel reflects each facet and is the average of all the items. The results of this analysis revealed a good level of model adjustment in the American and Basque samples: χ^2^_(69)_ = 96.20, *p* < 0.05; CFI = 0.98; TLI = 0.97; SRMR = 0.040; RMSEA = 0.036 (90% CI = 0.016–0.053) and χ^2^_(67)_ = 130.06, *p* < 0.05; CFI = 0.94; TLI = 0.91; SRMR = 0.046; RMSEA = 0.049 (90% CI = 0.036–0.061), respectively.

#### Structural Model: Partial Versus Full Mediation

Structural equation models (SEM) using maximum likelihood estimations were conducted to examine the mediating effect of resilience on the influence of EI on perceived stress. To verify the relationships among all the variables analyzed in this study, the values of goodness-of-fit indices obtained from the hypothesized model of partial mediation and the model of full mediation for the American and Basque samples were compared (see Table 3). On the one hand, the partial mediating model tested the possible direct effect of EI on perceived stress, and, on the other, the full mediating model constrained this potential direct path to zero.

**Table 3:** Model fit summary for the final structural model for the American and Basque samples.

Fit index	Suggested value Hair et al. (2010)	American sample		Basque sample	
		Hypothesized model	Alternative model	Hypothesized model	Alternative model
χ^2^	*p* < 0.05	96.20	107.79	120.01	130.06
df	n/a	69	70	69	67
χ^2^/df	<5 preferably <3	1.39	1.54	1.74	1.94
CFI	>0.90	0.98	0.97	0.94	0.93
TLI	>0.90	0.97	0.96	0.93	0.91
SRMR	<0.10	0.040	0.044	0.059	0.046
RMSEA_(IC)_	<0.08	0.036_(0.016-0.053)_	0.042_(0.026-0.058)_	0.043_(0.030-0.056)_	0.049_(0.036-0.061)_
ECVI_(IC)_		0.59_(0.51-0.70)_	0.56_(0.49-0.66)_	0.51_(0.45-0.61)_	0.52_(0.45-0.62)_

**FIGURE 2:**
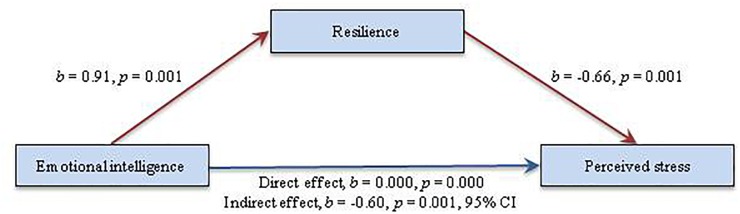
Standardized Solution of the Final Model in the American Sample.

Based on the same criteria used for evaluating the relationships between the different constructs involved in the research, the results of the proposed structural model for the American and Basque samples were very close together. With regards to the American sample, the hypothesized model (partial mediating model) fit the data adequately: χ^2^_(69)_ = 96.20, *p* < 0.05; CFI = 0.98; TLI = 0.97; SRMR = 0.040; RMSEA = 0.036 (90% CI = 0.016–0.053). Nevertheless, after a detailed examination of the estimated parameters it was noted that the path from resilience to perceived stress (β = -0.147, *p* = 0.700) did not provide the expected significant explanatory level. These results suggested that resilience is not directly associated with perceived stress when a direct path from EI to perceived stress is included in the model. In addition to testing the indirect effect of EI on perceived stress through resilience, the alternative model (full mediating model) also suggested an adequate global fit: χ^2^_(70)_ = 107.79, *p* < 0.05; CFI = 0.97; TLI = 0.96; SRMR = 0.044; RMSEA = 0.042 (90% CI = 0.026–0.058). In view of the regression coefficients, all of the suggested pathways achieved statistically significant levels. Considering the contrast between two models, as well as the degrees of freedom, the chi-squared test [Δχ^2^_(1,300)_ = 11.59, *p* < 0.001] corroborated the significant difference between the tested models. The cross-validation index (ECVI) also supported the fact that both models were significantly different; besides, it confirmed that the alternative model showed more replicability due to its lower value as compared to the index obtained in the hypothesized model. As a consequence, the full mediating model concerning the relationship between EI and perceived stress was supported. Standardized path coefficients for the final mediational model in the American sample are depicted in Figure 2.

**FIGURE 3:**
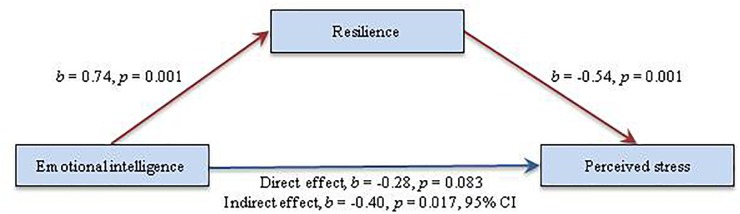
Standardized Solution of the Final Model in the Basque Sample.

As regards to the Basque sample, the resulting parameters obtained in the hypothesized model (partial mediating model) were within accepted limits, and thus suggested an acceptable global fit: χ^2^_(69)_ = 120.01, *p* < 0.05; CFI = 0.94; TLI = 0.93; SRMR = 0.059; RMSEA = 0.043 (90% CI = 0.030–0.056). Excepting the path from EI to perceived stress (β = -0.277, *p* = 0.083), all the proposed directions obtained significance level (*p* < 0.001). Accordingly, there seems to be no direct link between these variables in the proposed model. Besides that, the alternative model (full mediating model) also showed a good global fit: χ^2^_(67)_ = 130.06, *p* < 0.05; CFI = 0.93; TLI = 0.91; SRMR = 0.046; RMSEA = 0.049 (90% CI = 0.036–0.061). After analyzing the estimated parameters, all the suggested paths were found to reach statistically significant levels (*p* < 0.001). The comparison between the models using the chi-square difference test indicated that the two models were significantly different [Δχ^2^_(1,398)_ = 10.05, *p* < 0.001]. This was also verified by the ECVI, according to which the hypothesized model (Figure 3) presents a better degree of replicability compared to the alternative mediating model.

#### Path Coefficients and Assessment of Mediation

The significance of the mediating effect of resilience in both final models was assessed using the non-parametric Bootstrap procedure in AMOS 24. Specifically, 2000 bootstrap iterations were generated though random sampling with replacement from the data set in each sample (*N*_Americansample_ = 300; *N*_Basquesample_ = 398).

Standardized direct and indirect effects of EI and resilience on perceived stress are presented in Table 4. If the model’s regression coefficients are analyzed individually, it is clear that the majority of the supposed direct effects obtained significant levels (*p* < 0.001; *p* < 0.05), with the exception of the EI-perceived stress pathway in the Basque sample (β_Basquesample_ = -0.28, *p* > 0.05). EI revealed a significant positive path to resilience (β_Americansample_ = 0.91, *p* < 0.001; β_Basquesample_ = 0.74, *p* < 0.001), showing a statistically significantly higher regression coefficient in the American sample compared to the Basque sample.

**Table 4:** Model pathways, bootstrapped point estimates of direct and indirect effects, *p*-values and study results.

Hypothesis	Model pathways	Point estimate	*p*-value	Study results
**American sample**				
Hypothesis 1	EI → RE direct	0.912***	0.001	Supported
Hypothesis 2	RE → PS direct	-0.663***	0.001	Supported
Hypothesis 3	EI → PS direct	0.000	0.000	Not supported
Hypothesis 4	EI → PS indirect	-0.605***	0.001	Supported
**Basque sample**				
Hypothesis 1	EI → RE direct	0.741***	0.001	Supported
Hypothesis 2	RE → PS direct	-0.544***	0.001	Supported
Hypothesis 3	EI → PS direct	-0.277	0.083	Not supported
Hypothesis 4	EI → PS indirect	-0.403*	0.017	Supported

*^∗^p < 0.05, ^∗∗∗^p < 0.001. EI, emotional intelligence. RE, resilience. PS, perceived stress.*

On the other hand, a significant path from resilience to perceived stress could be observed, showing a higher negative coefficient in the American sample (β_Americansample_ = -0.66, *p* < 0.001; β_Basquesample_ = -0.54, *p* < 0.001).

According to the results, the effect of EI on perceived stress was completely mediated by resilience in both samples (β_Americansample_ = -0.60, *p* < 0.001; β_Basquesample_ = -0.40, *p* < 0.05), explaining the 44 and 60% of the variance in perceived stress in the American and Basque sample, respectively. To see the path coefficients of the ultimate model in standardized form, see Figures 2, 3.

## Discussion

The main goal of this cross-country study was to simultaneously evaluate the association between EI, resilience and perceived stress in two samples of undergraduate students. Congruent with our primary hypothesis, we found that the previously reported positive effect of EI on resilience (Armstrong et al., 2011; Cejudo et al., 2016; Magnano et al., 2016) was significant in the two countries -the Basque Country and the United States-. These findings revealed that the ability of undergraduate students to identify and manage their own emotions, as well as other’s emotions seems to have a predictive impact on their ability to cope with developmental tasks despite the risks.

Consistent with our second hypothesis, resilience was expected to have a significantly negative impact on perceived stress. Findings did confirm this hypothesis in Basque and American students. This is in accordance with previous studies that suggest that individuals with high resilience may recover effectively from daily stress (Ong et al., 2006). Likewise, studies report resilience as a crucial source of students’ healthy adaptation despite difficult or unpleasant situations (Wright et al., 2013; Rodríguez-Fernández et al., 2016). Some recent research looks at the buffering impact of resilience on daily stressors leading to lower psychological discomfort (McKay et al., 2018); however, few studies look at the processes underlying such findings.

Furthermore, contrary to our third hypothesis, we found that the models of the current study did not support the negative direct impact from EI to perceived stress. Based on the results obtained in studies that have evaluated the relationship between EI and perceived stress (Jung et al., 2016; Urquijo et al., 2016; Zysberg et al., 2017), we hypothesized that EI would be negatively associated with perceived stress. However, results have shown that there is not a direct link between these two variables, but an indirect one (EI and perceived stress are connected through resilience). Our results are in line with those of Zeidner and Olnick-Shemesh (2010), which posit that emotionally intelligent individuals may have superior skills in coping with threatening events, either through direct management of stressors, or through finding opportunities for personal growth and learning in adverse situations. Thus, it is suggested that there could be some different possible pathways for psychological impacts on adaptative responses. Our results demonstrated evidence of an indirect role for EI when individuals possess resilience characteristics that protect them from stress. Indeed, EI may lead individuals to become more resilient, indicating that resilience is not only the complement of psychological readjustment, but more (Ong et al., 2006).

In line with our expectations in relation to our fourth hypothesis, the specific indirect effect of EI on perceived stress via resilience was significant in both samples. That is, undergraduate students with higher levels of EI were inclined to be more resilient, which may contribute to a decrease in perceived stress. If we compare the input of the different variables included in the theoretical model, it becomes clear that perceived stress is indirectly determined by EI. Moreover, and in accordance with Hodzic et al. (2016) we identify that EI, as a protective factor promoting stress-resiliency, plays a paramount role in the activation of strategies that could help in protecting psychological adjustment.

Regarding the fifth hypothesis, bearing in mind that the association between EI-resilience-perceived stress has been the same in both countries, hypothesis five has been confirmed. These results are in line with those cross-cultural/country studies that show that there are differences between European–American and Eastern Asian cultures, but not between European and American cultures (Gökçen et al., 2014; Li and Yang, 2016; Nozaki, 2018). Given that our study includes one European sample and one American, we confirm the results of those studies that have not found differences between these cultures.

This research offers remarkable strengths, such as the cross-country nature of the study, and the assessment of the way EI affects perceived stress by analyzing the potential mediating influence of resilience. Regarding the cross-country nature of this research, it must be highlighted that although EI, resilience and stress have already been examined in other studies, there is little evidence from cross-country studies of the simultaneous relationship between these psychological variables. As for the further knowledge obtained regarding the association between EI, resilience and perceived stress, the study has shed light on the way EI affects perceived stress by analyzing the potential mediating influence of resilience, thus bridging the gap in relation to previous research. In this regard, the most important contribution of this study is that resilience is a mediator of the relationship between EI and perceived stress in young students. These results allow us to strongly suggest that resilience plays a crucial role in determining psychological health during university years.

As future lines of research, it is suggested to replicate this investigation using other potential mediators that might act between EI and perceived stress, as well as explaining the relationship between these variables. It has been demonstrated that EI is related to subjective well-being, mental health, social support, etc.; that resilience is associated to well-being outcomes like satisfaction with life, affect, self-concept and engagement; and that stress is related to low self-esteem, little optimism and a low sense of self-efficacy, among others. Thus, subjective well-being, self-esteem, satisfaction with life or self-concept could be potential mediators acting between EI and perceived stress.

The results obtained in the study have important applied implications. In fact, it would be beneficial for practitioners to recognize that EI and resilience may be used to prevent or reduce perceived stress. Thus, intervention programs that improve both EI and resilience could be helpful in reducing perceived stress. Such interventions could be applied in universities to develop EI and resilience, and avoid perceived stress. In fact, undergraduate students suffer from high perceived stress levels, and it would be helpful for them to develop their EI and resilience, thus reducing their perceived stress and improving their mental health.

A few limitations of this study warrant mention. First, it should be noted that in both samples women outnumber men. This gender imbalance represents the existing reality in certain university degrees (such as Psychology and Education) in which objectively there is a higher percentage of female students. Nevertheless, in future studies an attempt could be made to achieve a balanced participation of both genders, by collecting data in university degrees in which there is a higher proportion of male students or in which both subgroups are more balanced. Second, it should be made clear that due to the design used this study proves relations and predictive capacity between variables; but never shows cause-effect associations. Therefore, further research to test the model with longitudinal data to establish the casual relationships. Third, it should be mentioned that the sample of the study is imbalanced regarding the degree courses. In fact, the participants of the present study come from Psychology and Education, two similar degrees that are not representative of the variety of university courses. Thus, conclusions should be interpreted with caution. Fourth, the length of the PSS-4 should be highlighted. Although it has good psychometric properties (good reliability and validity), perceived stress has been evaluated using only four items. It would be interesting for future studies to replicate the study using longer versions of the PSS in order to verify the results. Fifth, the small size of the sample should be highlighted. As the sample of the study is comprised with 300 undergraduates from the University of Nevada (United States) and 398 from the University of the Basque Country, we cannot generalize the results to the general population of the United States and Basque Country as 300 and 398 are not representative of the whole population. Sixth, and connected with the aforementioned limitation, we should clarify that although we have tried to fill the gap in cross-country studies regarding EI, resilience and perceived stress, more research is needed in order to fill the gap. In fact, the present study is a first step that should be followed by more research to deepen in the field. Seventh, the low internal consistency of the Facilitation of emotions subscale should also be mentioned. In fact, this score may not be enough (Nunnally, 1978) due to the fact that a significant part of the variance would be explained by other factors, putting the internal reliability of the results obtained in this subscale at risk. The low internal consistency could be explained by the fact that Facilitation of emotions subscale consists in only three items. Nevertheless, as the total SREIS presents good internal consistency, the results presented in the study are reliable. Finally, future research may shed more light on the mediational variables between EI and perceived stress by integrating other psychological and contextual variables.

## Conclusion

The results of this cross-national study demonstrate a predictive effect of EI and resilience on stress. Resilience can be considered a skill. Likewise, it has been proved that EI can be trained and thus improved (Mikolajczak and Peña-Sarrionandia, 2015). In this way, this study may easily conclude that EI and resilience would buffer the negative influence of stress. Therefore, it is essential to design socio-emotional intervention programs whose objective is to enhance these psychological variables (EI and resilience). Only through the implementation of such interventions under an experimental design and a longitudinal study will it be possible to verify whether the improvement of EI influences the levels of resilience and whether these, in turn, have an impact on stress reduction in undergraduate students.

## Author Contributions

ER-D collected data in the United States. ER-D, AS, and OF-L collected data in the Basque Country. ER-D ran the statistical analysis and wrote the methodological part. OF-L helped in the methodological part. AS and ER-D wrote the introduction. AS, ER-D, and OF-L wrote the discussion and revised the paper regarding the contents. AS revised the paper regarding the format.

## Conflict of Interest Statement

The authors declare that the research was conducted in the absence of any commercial or financial relationships that could be construed as a potential conflict of interest.
